# OGG1 Preserves Endothelial-Dependent Vasodilation and Regulates the Frequency and Spatial Area of Endothelial Calcium Signals

**DOI:** 10.3390/biom15060790

**Published:** 2025-05-29

**Authors:** Takreem Aziz, Larysa Yuzefovych, Lyudmila Rachek, Mark S. Taylor, Christopher M. Francis

**Affiliations:** 1 Department of Physiology and Cell Biology, University of South Alabama College of Medicine, Mobile, AL 36688, USA; 2 Department of Pharmacology, University of South Alabama College of Medicine, Mobile, AL 36688, USA

**Keywords:** base excision repair, endothelial function, calcium signaling, myography, confocal imaging, vasodilation, endothelial phenotype, OGG1

## Abstract

Endothelial calcium dysregulation underlies impairments in endothelial-dependent vasodilation (EDV), contributing to vascular disease progression. Repletion of 8-oxoguanine DNA glycosylase (OGG1), an enzyme involved in base excision repair, has been shown to forestall vascular disease progression. However, the role of OGG1 in regulating endothelial calcium dynamics and in preserving EDV is unknown. Here, calcium imaging via high-speed confocal microscopy and automated analytics was used to quantify the spatial and temporal parameters of endothelial calcium signals in the excised carotid arteries of male and female C57BL6J/FVBNJ mice aged 4–7 months with normal endogenous levels of OGG1, in mice lacking OGG1, and in mice with repleted human OGG1 targeted to the mitochondria. Mice lacking OGG1 exhibited an anomalous calcium phenotype characterized by a substantial increase in the basal tissue-wide frequency and spatial area of the endothelial calcium signals. Mitochondrial repletion of hOGG1 restored the calcium phenotype under unstimulated and acetylcholine-stimulated conditions. EDV was assessed using pressure myography. Mice lacking OGG1 exhibited significant impairments in EDV in response to acetylcholine, and the mitochondrial repletion of OGG1 rescued EDV. These findings highlight a novel role for OGG1 in endothelial signaling and suggest its importance in vascular homeostasis.

## 1. Introduction

The endothelium regulates vascular tone, largely through the production and release of both vasodilatory and vasoconstrictive substances [1]. The major vasodilator substance released by the endothelium is nitric oxide (NO), synthesized by endothelial nitric oxide synthase (eNOS) [2]. Dynamic changes in intracellular calcium are a critical mechanism governing endothelial cell phenotype, NO production, and vascular smooth muscle cell (VSMC) contractility, ultimately enabling EDV [1,3,4,5,6]. Impaired EDV, largely due to a reduction in NO bioactivity and bioavailability during oxidative stress, is a major facet of the pathogenesis of vascular disease [2,7,8].

OGG1 is a key base excision repair (BER) enzyme that removes 8-oxoguanine (8-oxoG), an injurious DNA base lesion caused by oxidative stress [9,10]. OGG1 is pivotal for preventing the subsequent genomic instability and cellular dysfunction associated with 8-oxoG accumulation. Loss of OGG1 is associated with the increased severity of vascular disease. Targeted deletion or knockdown of *Ogg1* enhances 8-oxoG accumulation, as well as increases cell death, senescence, and the expression of inflammasome components, all of which are proatherogenic [11]. In the absence of OGG1, mice fed a Western diet exhibit significantly increased atherosclerotic lesion areas and macrophage content [12]. Finally, the absence of OGG1 lowers arterial compliance and distensibility to promote vascular stiffness in aging, ultimately contributing to atherosclerosis [13]. Mitochondrial DNA is highly susceptible to oxidative damage due to its proximity to the electron transport chain and lack of protective histones. Unlike nuclear DNA, mitochondria rely solely on BER, making OGG1 in this organelle particularly relevant.

Overexpression of *Ogg1* has been shown to confer protection against cardiovascular disease progression. Overexpression of *Ogg1* significantly reduces apoptosis in the pulmonary arterial endothelial cells of mice fed with a Western diet, suggesting it as a key player in forestalling atherosclerosis [12,14]. Notably, overexpressing human OGG1 targeted to the mitochondria in H9C2 cells greatly improves mitochondrial function and cell survival, while also lowering mitochondrial (mtDNA) deletions through an increase in BER activity under oxidative stress conditions [15]. Mitochondrial OGG1 overexpression decreases mtDNA oxidation, restores mtDNA copy number, and blunts elevations in cardiac mass, reducing cardiac pathology [16]. In mice consuming a high fat diet, this overexpression lowers mitochondrial dysfunction and preserves cardiac contractility [16].

Finally, dysfunctional OGG1 is involved in the development of several diseases, including various forms of cancer [17,18,19] and neurological diseases [20,21]. Additionally, OGG1 deficiency contributed to the development of obesity and metabolic syndrome in mice [22]. A study of Vartainian et al. showed that OGG1-deficient mice display increased ectopic lipid accumulation in the skeletal muscle, associated with increased mitochondrial fission and accelerated muscle function decline [23].

Although OGG1 has been studied in the context of vascular disease, its role in regulating endothelial calcium dynamics and vascular function through EDV, two critical determinants of endothelial health, is unknown. In this study, we implicated OGG1 as a target in modulating endothelial calcium dynamics and determined that OGG1 preserved the EDV of excised carotid arteries. These findings demonstrate an interplay between endothelial function and OGG1 and suggest that OGG1 may play a crucial role in preserving vascular homeostasis through the control of endothelial Ca^2+^ signaling.

## 2. Materials and Methods

Animal Usage: Three genotypes of mice bred with the mixed C57BL6J/FVBNJ background were utilized throughout the study—wildtype (WT), *Ogg1*−/− (*OGG1-KO*), a knockout strain void of *Ogg1*; and *Ogg1-KO*/*Tg* (a transgenic strain with repleted human OGG1 in the mitochondria attributable to a mitochondrial localization sequence). *OGG1-KO* mice were generated, as previously described, via targeted disruption of the murine *Ogg1* gene in embryonic stem cells [24]. Sequences encoding the highly conserved helix–hairpin–helix motif, required for enzyme activity, were replaced by Neo in the targeting vector. Transgenic mice with repleted human OGG1 in the mitochondria were generated previously as follows: the human OGG1 gene was fused to a mitochondrial translocation signal, provided with a specific Kozak sequence, and placed under the control of a CMV/AG promoter. Positive carriers from pronuclei injection (Norsk Transgen Senter) were crossed with previously generated *OGG1-KO* mice. Resulting mitochondrial OGG1 carriers were identified using PCR amplification [25]. All animals were bred at the University of South Alabama. Mice from all three groups were fed a standard diet (20% protein, 16% fat, 64% carbohydrates) for at least 12 weeks before usage and included both males and females. No restrictions were imposed on the amount of food or water consumed over the feeding period. Animal rooms were maintained at a temperature of 68–72 °F, with 12 h light/dark cycles. When intended for usage, the mice were euthanized via carbon dioxide exposure before undergoing any tissue harvesting protocols. All animals were genotyped prior to usage. All animal procedures were conducted upon approval of and in accordance with the IACUC of the University of South Alabama.

Tissue Isolation and Preparation: Common carotid arteries (~350–500 µm diameter) were surgically isolated. Upon isolation, carotid arteries were placed in 2-[4-(2-Hydroxyethyl) Piperazin-1-yl] Ethane Sulfonic Acid (HEPES) buffer, prepared in advance (134.0 mM NaCl, 6.0 mM KCl, 1.0 mM MgCl_2_, 10.0 mM Glucose, 0.03 mM EDTA, 2.0 mM CaCl_2_, 10.0 HEPES), for imaging and myography experiments.

Calcium Imaging: Cal-520 (AAT Bioquest, Pleasanton, CA, USA, 50 µg) was used as the fluorogenic calcium-sensitive dye of choice due to its improved signal-to-noise ratio and intracellular retention as compared to the abilities of the current calcium indicators. Surgically isolated carotid arteries were cut open lengthwise using micro scissors and planted onto Sylgard blocks using insect pins (Indigo Instruments). Sylgard 184 (Dow) was prepared beforehand by thoroughly mixing the base and curing agent, provided by the manufacturer, in a ratio of 10:1 by volume. The mixture was then left to cure for approximately 3 days at room temperature (20–23.3 °C). As per the manufacturer’s protocol, 50 µL of dimethyl sulfoxide (DMSO) was added into the 50 µg vial of Cal-520 and fully vortexed using a vortex mixer (Fisher Scientific standard vortex mixer). Additionally, in order to ensure that the Cal-520 was fully solubilized and able to effectively penetrate the endothelial cells, Pluronic F-127 (AAT Bioquest) was incorporated into the calcium dye mixture before incubation. In a 1 mL Eppendorf tube, 4 µM of Cal-520 and a 0.04% Pluronic F-127 working solution in HEPES buffer were vortexed thoroughly before being transferred into an empty, standard cell culture dish. The isolated carotid artery was then allowed to incubate within the dish, endothelial side down, for approximately 45 min before a 5 min wash in HEPES buffer and imaging via an Andor Revolution spinning disk confocal system (inverted microscope, ×20 objective magnification) using iQ software v3.6.1 (1024 × 1024 pixels, 488 nm excitation, 510 nm emission). The endothelial plane was determined by identifying the most intimal side of the pinned vessel, the endothelial cell morphology, and the perpendicular orientation to the underlying smooth muscle. The focal plane was then manually set to the endothelial field.

Calcium imaging included two measurement periods, one in the absence of exogeneous stimulation and a subsequent stimulated condition, administering successive concentrations of Acetylcholine. During imaging, time-lapse measurements were recorded for a total of five minutes, without interruption or stoppage. The first two minutes were dedicated for baseline recording. After two minutes, while recording was in progress, a concentration of ACh was administered. Three concentrations of ACh (1 pM, 1 nM, and 1 µM) were utilized, derived from dose response studies where 1 pM was the threshold concentration, 1 nM was the EC50, and 1 µM induced maximal dilatory responses. Importantly, if administration of ACh led to any alteration in the focal plane or a disruption of focus, the confocal microscope was adjusted immediately, and the specific, defective portion of the recording was either co-registered or excluded using Image J, a Java-based image processing program (National Institutes of Health). This process was repeated sequentially for the remaining ACh concentrations. All resulting calcium imaging data and measurements were analyzed using S8, a validated image processing algorithm for the analysis of second messenger signals [24]. After analysis, quality control and additional filtering was performed to ensure that the binarized image sequence reflected the true signal activity in the original image sequence and that any smooth muscle signals identified as perpendicular to the longitudinal endothelial direction were manually removed from the analysis of the signal frequency and area.

S8 Analysis Algorithm: Each time-lapse recording was collected as an image sequence and analyzed using S8 (version S8_23.05.31_DF_exp) to perform automated time-dependent region-of-interest measurements. Briefly, S8 performed time-series denoising using the Savitzky–Golay filter and minimum intensity background subtraction, followed by Otsu or Yen binarization. Lastly, the bitonal image was used to generate an image mask, and 3D particles comprising calcium signals were identified as dynamic (time-dependent) polygonal regions of interest, in which tissue-wide frequency and both cellular and tissue-wide spatial area measurements were collected. This analysis algorithm was published and validated using synthetically generated noisy datasets, enabling the decoding of signaling patterns in diverse tissues, along with the identification of pathologic cellular responses for signal-to-noise ratios near 1 [26].

Pressure Myography: Carotid arteries were transferred into the bath of the pressure myograph chamber (Danish Myo Technology (DMT) Myograph Chamber) containing 10 mL of HEPES buffer, formulated for pressure myography [27]. Before mounting, the myograph chamber was primed and flushed with HEPES buffer. Artery segments were mounted onto glass cannula tips and tied using surgical sutures.

A level of 65 mm Hg was achieved, in part, by using a traditional gravity-fed apparatus. The intravascular pressure was increased to 65 mm Hg and kept constant throughout the course of the experiment. The myograph chamber was mounted on a microscope equipped with live video recording abilities, and the diameter of the vessel was measured using DMT MyoView Vol 1.0 software (Danish Myo Technology).

Changes in the outer arterial diameter were assessed over successive time intervals. The first interval involved obtaining a baseline recording of the outer diameter of the artery. This was followed by fully pre-contracting the artery using a potent vasoconstrictor, U46619, (60 µM). Upon stable contraction, each successive interval involved administering acetylcholine (Ach), with gradually increasing concentrations (1 pM, 1 nM, and 1 µM). Following ACh response measurements, the carotid arteries were exposed to sodium nitroprusside, SNP, (10 µM) to assess direct responses to nitric oxide. All vasodilatory responses to ACh were expressed as percent maximal dilation, based on the total dynamic range of vasodilation and vasoconstriction that could be achieved by the carotid, using the following equation:

(1) $$\frac{100\times (Maximum\;Relaxation[SNP]-Response\;to\;[Ach])}{\left(Maximum\;Relaxation\left[SNP\right]-Max\;Contraction\left[U46619\right]\right)}=\%\;Maximum\;Dilation$$

Lastly, to ensure that our observed vasodilatory responses were indeed endothelium-dependent, we unfastened one end of the artery, physically denuded the endothelium by scraping it against the glass canula, and mounted the artery once more for final outer diameter measurement following a final administration of 1 µM ACh. Post-endothelial responses were expressed as the percentage change relative to pre-contracted diameter.

Statistical Methods: Calcium imaging experiments under baseline conditions were analyzed using one-way ANOVA with a Tukey HSD post hoc test, while calcium imaging experiments in the presence of ACh were analyzed using two-way ANOVA with a Tukey HSD post hoc test. Data acquired from the pressure myography experiments were analyzed using two-way ANOVA with a Tukey’s honestly significant difference (Tukey HSD) post hoc test. Data from U46619 pre-contraction was assessed using two-way ANOVA with a Sidak post hoc test, while post-endothelial denudation data were analyzed using one-way ANOVA. An alpha level of 0.05 was chosen to determine significant differences. The results are presented as mean ± standard deviation (SD).

## 3. Results

### 3.1. Loss of OGG1 Alters Endothelial Calcium Signaling Dynamics

**Figure 1:**
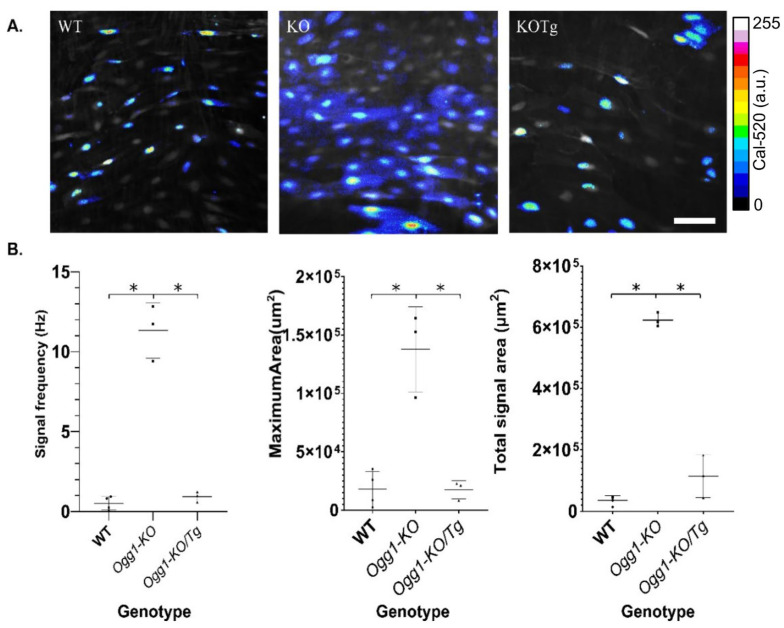
Analysis of endothelial calcium signaling phenotype. (**A**) Time-lapse measurements of carotid artery calcium activity from WT mice, *OGG1-KO* mice, and *Ogg1-KO*/*Tg* mice with repleted hOGG1 in the mitochondria. Carotid arteries from WT mice exhibited cell-wide calcium signals. Mice lacking OGG1 exhibited a distinct pattern, characterized by substantially higher activity and larger cumulative spatial area of calcium signals. *Ogg1-KO*/*Tg* mice with repleted hOGG1 normalized the calcium phenotype to that of the WT group. Pseudo-color areas indicate the location of dynamic calcium signals, as detected by automated analysis. Color scale indicates Cal-520 fluorescence intensity range in arbitrary units (a.u.). Scale bar denotes 5 µm. (**B**) Scatter plots showing frequency (Hz), cellular maximum, and total spatial area (µm^2^) of recorded calcium signals in WT mice, *OGG1-KO* mice, and *Ogg1-KO*/*Tg* mice. * Denotes a statistically significant difference from control (*p* < 0.0001). Results are presented as mean ± SD; *n* = 3–4.

Using the en face technique, carotid artery endothelial calcium activity was measured during a baseline interval, and the signal frequency of the tissue-wide calcium signal transients was analyzed. This analysis included the calculation of the spatial and temporal parameters associated with the calcium signal activity in each measurement interval. Signal frequency was defined as the tissue-wide number of signal transients at distinct cellular sites per second (Hz), reflecting the total amount of calcium activity. Analysis of the area encompassing each calcium signal utilized both the maximum signal area at each cellular signal transient location and the cumulative result of the spatial areas encompassed by all calcium signals within the tissue. These metrics reflect both the size of each individual signal transient, encompassing both subcellular and multicellular activity, and the total area of all calcium activity within the image sequences. Time-lapse summed images revealed striking differences in the calcium signaling phenotype dependent on OGG1 (Figure 1A). Carotids from the *OGG1-KO* group exhibited an anomalous calcium phenotype characterized by an increase in calcium signal frequency (11.3 ± 1.74 Hz vs. 0.51 ± 0.43 Hz), maximum per site (1.4 × 10^5^ µm^2^ vs. 1.8 × 10^4^ µm^2^), and total spatial area (6.2 × 10^5^ µm^2^ vs. 3.5 × 10^4^ µm^2^) compared to the results for the controls, respectively (*p* < 0.0001). Repletion of OGG1 (*Ogg1-KO*/*Tg*) rescued the calcium signal phenotype relative to wild type controls (Figure 1B).

### 3.2. OGG1 Regulates the Frequency and Spatial Area of Calcium Signals in the Presence of ACh

**Figure 2:**
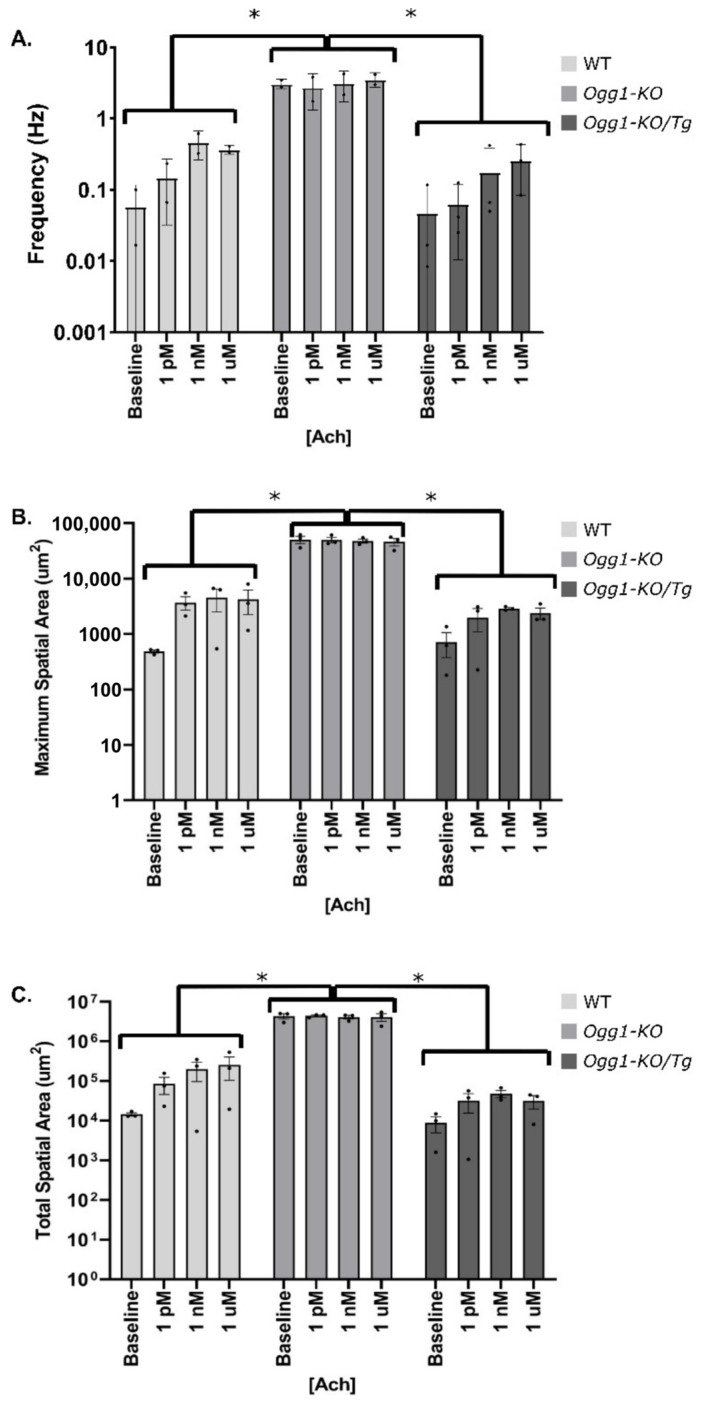
Analysis of endothelial calcium frequency and spatial spread in the presence of ACh. (**A**) Tissue-wide frequency (Hz) of calcium signals in response to sequential administration of three ACh concentrations (1 pM, 1 nM, and 1 µM) in WT mice, *OGG1-KO* mice, and *Ogg1-KO*/*Tg* mice with repleted human OGG1 in the mitochondria. *Y*-axis is represented in log scale. * Denotes a statistically significant difference from control (*p* = 0.0007). Results are presented as mean ± SD; *n* = 3. (**B**) Maximum spatial area (µm^2^) of calcium signals in response to sequential administration of three ACh concentrations (1 pM, 1 nM, and 1 µM) in WT mice, *OGG1-KO* mice, and *Ogg1-KO*/*Tg* mice with repleted OGG1. *Y*-axis is represented in log scale. * Denotes a statistically significant difference from control (*p* < 0.0001). Results are presented as mean ± SD; *n* = 3–4. (**C**) Total Spatial area (µm^2^) of calcium signals in response to sequential administration of three ACh concentrations (1 pM, 1 nM, and 1 µM) in WT mice, *OGG1-KO* mice, and *Ogg1-KO*/*Tg* with repleted OGG1 in the mitochondria. *Y*-axis is represented in log scale. * Denotes a statistically significant difference from control (*p* < 0.0001). Results are presented as mean ± SD, *n* = 3.

Sequential administration of ACh increased calcium signal frequency in the WT carotid artery endothelium. Arteries lacking OGG1 exhibited a significant increase in calcium signal frequency (*p* = 0.0007) as compared to that of the controls in the absence of Ach, which persisted under ACh-stimulated conditions. This difference in calcium signal frequency was restored between the WT and *Ogg1-KO*/*Tg* group in the presence of ACh (*p* > 0.9999) (Figure 2A). The absence of OGG1 significantly increased both the maximum (*p* < 0.0001) and total spatial area (*p* < 0.0001) of calcium activity in the presence of successive concentrations of ACh. Both the maximum and total spatial area of the *Ogg1-KO*/*Tg* group were rescued relative to those of the WT group (*p* > 0.9999) (Figure 2B,C).

### 3.3. Endothelial-Dependent Vasodilation Is Impaired by Loss of OGG1 and Rescued by Mitochondrial OGG1 Repletion

**Figure 3:**
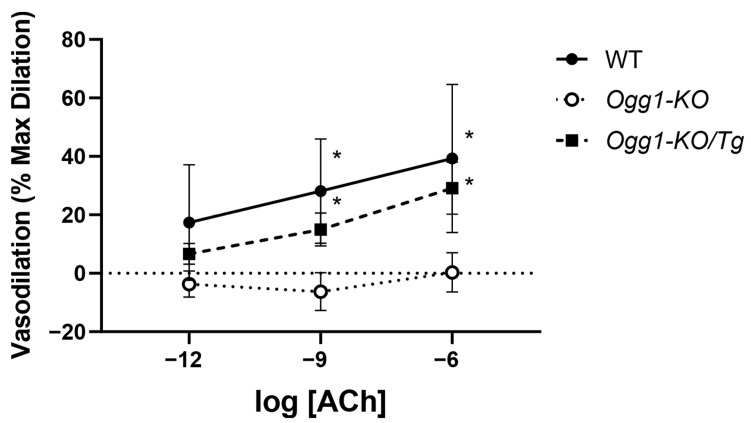
ACh-dependent vasodilation is impaired in OGG1-deficient mouse carotid arteries. Following pre-contraction by U46619 (60 µM), three concentrations of ACh (1 pM, 1 nM, 1 µM) were administered sequentially. Normalized dose-response vasodilation curves (% maximum dilation) are shown in response to sequential administrations of ACh in carotid arteries isolated from WT mice, *OGG1-KO* mice, and *Ogg1-KO*/*Tg* mice with repleted human OGG1 in the mitochondria. * Denotes a statistically significant difference from control. Results are presented as mean ± SD; *n* = 7.

Functional pressure myography performed on mounted mouse carotid arteries enabled the assessment of vasodilation in response to sequential concentrations of ACh after pre-contraction with U46619 (60 µM). Successive vasodilation was observed upon the administration of ACh in the wild-type (WT) group. Carotids from *OGG1-KO* group, however, showed significantly impaired vasodilation to ACh, often contracting in response to the successive ACh concentrations. The *Ogg1-KO*/*Tg* group with repleted human OGG1 in the mitochondria exhibited a rescued response to ACh that was indistinguishable from the WT. ACh-dependent relaxation was normalized to maximum diameter in response to SNP and minimum diameter following contraction by administration of U46619 (Figure 3).

**Figure 4:**
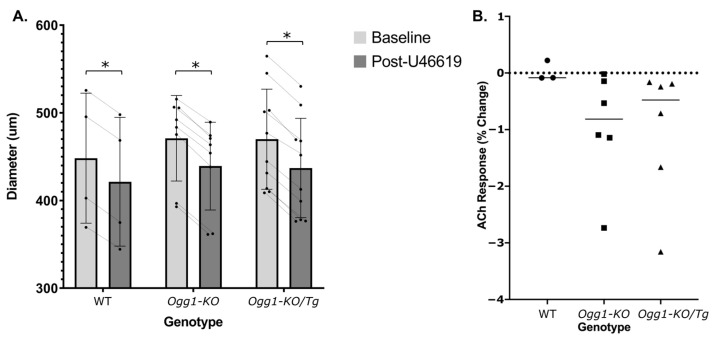
ACh responses are dependent on the endothelium. (**A**) Changes in arterial outer diameter in response to 60 µM U46619 administration across WT mice, *OGG1-KO* mice, and *Ogg1-KO*/*Tg* mice with repleted human OGG1 in the mitochondria. Bars on the left above each genotype group denote mean arterial outer diameter before U46619 administration while bars on the right above each genotype group denote mean arterial outer diameter post U46619 administration. * Denotes a statistically significant difference from control (*p* < 0.0001). Results are presented as mean ± SD; *n* = 4–10. (**B**) Responses to 1 µM ACh administration post-endothelial denudation across WT, *OGG1-KO*, and *Ogg1-KO*/*Tg* mice. Responses below a percent change of zero denote arterial contraction. All responses to ACh are represented as percent change; *n* = 3–6.

Administration of U46619 led to constriction in carotids isolated from all three groups (*p* < 0.0001) (Figure 4A). Notably, difference in genotype did not statistically alter the degree of U46619-induced constriction (*p* = 0.9098) indicating that the presence, absence, or repletion of OGG1 is not a determinant of contractile responses to the thromboxane analog, U46619 (Figure 4A). Finally, vasodilation in response to ACh post-endothelial denudation was non-existent and statistically insignificant between each of the three groups (*p* = 0.335), confirming that the observed ACh vasodilatory responses were indeed endothelial-dependent (Figure 4B).

## 4. Discussion

In this study, we found that the absence of OGG1 gives rise to a distinct, anomalous calcium phenotype characterized by substantially increased frequency and the spatial spread of calcium signals under both baseline and stimulated conditions and that repletion of human OGG1 in the mitochondria normalizes the aberrant calcium phenotype under both unstimulated and ACh-stimulated conditions. Furthermore, we offer evidence for a novel role of OGG1 in preserving EDV and modulating the frequency and spatial spread of endothelial calcium dynamics. Our results indicate that in the carotid endothelium, the absence of OGG1 translates to drastic impairments in the EDV as compared to that of the controls, and the repletion of hOGG1 in the mitochondria rescues those impairments.

Here, we utilized a transgenic mouse model with repleted human OGG1 in the mitochondria. Mitochondrial overexpression has been shown to be more impactful in terms of protection from oxidative DNA damage compared to the results for repletion in the nucleus. Much of this is accredited to the biological nature of mitochondria. For example, mtDNA is much more susceptible to oxidative base damage due to its proximity to the electron transport chain, the major source of reactive oxygen species (ROS) within the cell, and due to its lack of protective histones present in nuclear DNA [28]. Additionally, unlike the nucleus, which possesses both BER and nucleotide excision repair (NER), mitochondria can only utilize BER. This transgenic mouse model is well documented in the literature and has been shown to exhibit beneficial effects in several disease states, which are not limited only to vasculature diseases. Our findings build upon the concept of OGG1 playing a beneficial role in opposing vascular disease, particularly through regulating endothelial calcium dynamics and preserving EDV, two critical determinants of ensuring vascular tone.

Results derived from our calcium imaging experiments, under both baseline and stimulated conditions, offer evidence of a novel use for OGG1 in the management of endothelial calcium dynamics. Our data indicate that the absence of OGG1 gives rise to a calcium phenotype characterized by an increase in the frequency of calcium signals. While increased calcium signal frequency suggests heightened endothelial excitability, the expanded spatial area may indicate aberrant intercellular calcium propagation, possibly affecting the vessel-wide coordination of vasodilation. Implications of a high frequency calcium phenotype in vascular disease have been documented in the literature. For example, we previously reported a high-calcium frequency phenotype in a model of severe pulmonary arterial hypertension (PAH), suggesting that it underlies endothelial dysfunction in the pathophysiology of PAH [29]. Since alterations in intracellular calcium occur upstream of EDV, our results suggest that these observed phenotypic differences in endothelial calcium dynamics could be translated into the functional differences in EDV which we discovered in our pressure myography studies.

We further discovered that the absence of OGG1 led to substantial increases in both the maximum cellular and total tissue-wide spatial area of calcium signals. Unlike a high-frequency calcium phenotype, the implications of the increased spatial area of calcium signals are less clear. However, the maximum and total spatial area of calcium signals presented in our work are also important metrics that represent, and can be used to describe, intracellular and tissue-wide calcium levels. A possible underlying mechanism may be that an aberrant increase in ROS production alters the activity, expression, or open-time probability of several proteins involved in intracellular calcium regulation [30,31]. The consequence of such abnormal elevations in intracellular calcium is the induction of pro-apoptotic events and cell death via apoptosis, both of which are frequently present in vascular disease. For example, Thomas et al. demonstrated that apoptosis precedes the development of atherosclerotic plaques in swine fed a hypercholesterolemic diet [32]. Furthermore, Bennett et al. demonstrated that human atherosclerotic plaque-derived smooth muscle cells display a markedly elevated rate of apoptosis [33]. Lastly, Y.J. Geng and P. Libby revealed that VSMCs located in the intimal fibrotic portion of the atherosclerotic plaque show increased evidence of apoptosis as compared to the levels in the controls [34]. Whether OGG1 deficiency leads to endothelial apoptosis in vivo or the selection of an apoptotic resistant endothelial phenotype is unknown. Future studies should examine whether OGG1 deficiency increases endothelial apoptosis by assessing markers such as cleaved caspase-3.

Although our ACh administrations resulted in a largely sigmoidal dose-response across all three genotypes in our calcium imaging experiments, ACh did not significantly alter calcium dynamics, suggesting that OGG1’s effects on calcium signaling are independent of muscarinic receptor activation. A similar scenario was witnessed by McFarland et al., where they discovered that in an endothelium-specific TRPV4 knockout mouse model, ACh administration did not lead to a significant difference in spatial area as compared to that of WT mice, although an increase in spatial area was observed with each concentration [35]. Remarkably, however, the presence, absence, and repletion of *hOGG1* remained the defining factor influencing the calcium signal frequency and spatial area of our carotids.

OGG1 has been studied in the context of vascular disease. However, any evidence linking OGG1 to EDV is currently unknown. Our results are the first to offer evidence of a novel role for OGG1 in this regard. Our data indicated that the absence and repletion of OGG1 translated to substantial differences in EDV as compared to that of the controls. It is well established that the absence of OGG1 induces ROS production and subsequent oxidative stress [36,37,38]. For example, Bacsi et al. demonstrated that increased ROS production is strongly prevalent in mice fibroblasts in the absence of OGG1 [36]. Such increases in ROS production could underlie impairments in EDV due to degradation or loss of NO activity followed by eNOS uncoupling. Indeed, Thum et al. indicated that uncoupling of eNOS and subsequent eNOS-driven superoxide production is evident in the EPC from diabetic patients, thus contributing to the pathogenesis of vascular disease [39]. Moreover, Landmesser et al. discovered eNOS uncoupling and eNOS-driven ROS production in the aortas of mice with deoxycorticosterone acetate–salt induced hypertension [40]. While ROS production is implicated in endothelial dysfunction, direct ROS quantification was beyond the scope of this study.

OGG1 is also an epigenetic regulator of transcription via excision of 8-oxoG lesions in gene promoters, which can alter chromatin structure and recruit transcriptional co-activators or repressors [9,41]. Indeed, in mouse fibroblast, Ding et al. found 8-oxoG lesions within the genes of key calcium signaling proteins including Atp2a2, encoding the sarco/endoplasmic reticulum calcium ATPase (SERCA), and Itpr2, encoding inositol 1,4,5-trisphosphate receptor type 2 (IP_3_R2), that regulate the endothelial control of vascular tone [42]. We did not quantify OGG1 expression or activity, nor assess 8-oxoG occupancy at calcium-regulatory gene loci, potentially overlooking its impact on intracellular calcium dynamics. Studies including targeted analyses of the OGG1-dependent regulation of calcium-handling gene expression are essential for determining the role of OGG1 in the epigenetic regulation of vascular tone.

Upon assessing our post-endothelial denudation data, we confirmed that the vasodilatory responses were endothelial-dependent. Indeed, most carotid arteries contracted in response to ACh administration, which was expected, since it is well-established that in the absence of the endothelium, administration of ACh triggers endothelial-independent contraction via the activation of muscarinic receptors located within VSMCs, thus promoting contraction instead of vasodilation [43]. We also demonstrated that the effect of U46619 administration on arterial constriction was identical across all three mouse genotypes, indicating that the ability of the artery to contract is not dependent on OGG1.

Endothelial dysfunction and atherosclerosis are frequently observed in carotid artery disease [44,45]. Because of this, carotid arteries are extensively utilized in vascular studies. However, given that carotids are conduit arteries, their capacity to vasodilate and vasoconstrict in response to vasodilatory and vasoconstrictive agonists is limited as compared to that of resistance arteries such as the femoral or mesenteric arteries [27]. Although carotid arteries used in our pressure myography experiments were contracted fully before administering ACh, it was evident that their capacity to contract was not immense, i.e., 30–40 µm in the outer diameter.

## 5. Conclusions

In this study, we established that the BER protein OGG1 is important in the regulation of endothelial signaling and carotid vasoreactivity. We found that the presence of OGG1 is necessary to limit the compartmentalization of calcium signal transients, impairing endothelial capacity to regulate signal strength in an agonist-dependent manner. These findings add a new key determinant to the many regulators of calcium signaling—especially in the context of vascular physiology and disease.

Our usage of dynamic region-of-interest tracking software, S8, enabled the measurement of time-dependent changes in calcium signal area and frequency regulated by OGG1. However, due to the reliance on time-varying rather than static regions of interest, fluorescence intensity measurements, traditionally expressed as the fold change over the signal baseline, were not possible. Employing additional static region-of-interest approaches to complement our findings may be warranted.

While our experiments pooled data across sexes, it is established that sex differences in carotid vasoreactivity exist, especially in older mice. We did not stratify our analyses by sex nor control for the estrous cycle, potentially obscuring sex-specific effects. The absence of a sex-based analysis of calcium signal and vasodilatory responses could obscure biologically relevant differences. Future studies should include balanced cohorts of male and female mice, as acknowledging these sex differences is important for improving the translational relevance of the OGG1-dependent regulation of the vascular endothelium.

Because our studies were performed in excised arteries and not in pure cell cultures, reliably assessing NO and/or ROS production with difluorofluorescein diacetate, diaminorhodamine-4M, or MitoSox Red (Invitrogen) was not possible, despite multiple attempts. This limitation precluded direct mechanistic evidence of impairment of endothelial NO production. However, given that increased ROS production has been demonstrated to precede pro-apoptotic events and apoptosis, and given that such events precede vascular disease, assessing ROS production and evaluating endothelial cell death in the presence and absence of OGG1 is a significant future direction. Furthermore, we also deem it important to determine whether mitochondrial OGG1 repletion safeguards against potential increases in ROS production or prevents the induction of pro-apoptotic events or cell death. Given the role of endothelial dysfunction in atherosclerosis and stroke, further studies should explore whether OGG1 augmentation could serve as a therapeutic strategy for vascular disease.

## Data Availability

The original data presented in the study are openly available in Google Drive at https://drive.google.com/drive/folders/14LCPV-NuHO6kyubLtHSewjljLYyy5BUG?usp=sharing. (accessed on 28 February 2025).
